# Differential brain activity in patients with disorders of consciousness: a 3-month rs-fMRI study using amplitude of low-frequency fluctuation

**DOI:** 10.3389/fneur.2024.1477596

**Published:** 2024-12-13

**Authors:** Xuewei Qin, Xuanling Chen, Lan Yao, Fa Lu, Zhenhu Liang, Jianghong He, Xiangyang Guo, Xiaoli Li

**Affiliations:** ^1^Department of Anesthesiology, Peking University International Hospital, Beijing, China; ^2^Institute of Electrical Engineering, Yanshan University, Qinhuangdao, China; ^3^Department of Neurosurgery, Beijing Tiantan Hospital, Capital Medical University, Beijing, China; ^4^Department of Anesthesiology, Peking University Third Hospital, Beijing, China; ^5^State Key Laboratory of Cognitive Neuroscience and Learning, IDG/McGovern Institute for Brain Research, Beijing Normal University, Beijing, China; ^6^Center for Collaboration and Innovation in Brain and Learning Sciences, Beijing Normal University, Beijing, China

**Keywords:** disorders of consciousness, rs-fMRI, precuneus, cingulate gyrus, ALFF, spinal cord stimulation

## Abstract

**Introduction:**

Disorders of consciousness (DoC) from severe brain injuries have significant impacts. However, further research on nuanced biomarkers is needed to fully understand the condition. This study employed resting-state functional MRI (rs-fMRI) and the amplitude of low-frequency fluctuation (ALFF) to investigate differential brain activity in patients with DoC following spinal cord stimulation (SCS) therapy. It also assessed the predictive value of rs-fMRI and ALFF in determining the consciousness levels at 3 months post-therapy.

**Methods:**

We analyzed rs-fMRI data from 31 patients with traumatic brain injury (TBI) and 22 with non-traumatic brain injury (non-TBI) diagnosed with DoC. ALFF was measured before SCS therapy, and clinical outcomes were assessed 3 months later using the Coma Recovery Scale-Revised.

**Results:**

Patients with TBI showed increased ALFF in the thalamus and anterior cingulate cortex, whereas the middle occipital lobe showed decreased ALFF. In the non-TBI group, a higher ALFF was noted in the precuneus, with a reduced ALFF in the occipital and temporal lobes. Patients with improved consciousness post-SCS exhibited distinct ALFF patterns compared with those with unchanged consciousness, particularly in the posterior cingulate and occipital regions.

**Conclusion:**

The application of ALFF in rs-fMRI may be a predictive tool for post-treatment outcomes in patients with DoC of varying etiologies. Differential ALFF in specific brain regions could indicate the likelihood of improvement in consciousness following SCS therapy.

**Clinical trial registration:**

https://www.chictr.org.cn/, Identifier ChiCTR2300069756.

## 1 Introduction

Disorders of consciousness (DoC) are neurological disorders caused by traumatic brain injury (TBI), such as car accidents or falls, and non-traumatic brain injury (non-TBI), such as stroke, etc., and are characterized by decreased awareness and responsiveness. Patients with DoC may exist in states ranging from the unresponsiveness of a vegetative state to minimal yet discernible signs of consciousness found in minimally conscious states (MCSs) (1). These conditions are particularly challenging for clinicians because of the patient’s limited ability to interact with their environment, complicating the assessment of cognitive and sensory functions (2).

Traditional clinical evaluations rely on behavioral scales, like the Coma Recovery Scale-Revised (CRS-R), to provide a standardized method for evaluating the level of consciousness (3, 4). In 2004, the Coma Recovery Scale-Revised (CRS-R) was published, comprising six subscales that assess auditory, verbal, visual, communicative, motor, and arousal domains. The CRS-R includes 23 hierarchically structured items, with scores ranging from 0 to 23, with higher scores signifying greater levels of awareness and functional capacity (5, 6). However, subtle and sporadic responses in DoC can lead to diagnostic ambiguities and difficulties in predicting recovery. The need for more objective and sensitive measures has spurred the exploration of advanced neuroimaging techniques that offer insights into the functional architecture of the brain beyond the confines of behavioral observation (7).

In China, some medical units have prioritized research into wakefulness treatments of patients with DoC. SCS represents a neuromodulation therapy for patients diagnosed with DoC. The stimulation electrode is implanted at the C2–C4 level of the high cervical vertebrae through the epidural cavity. Sending electrical stimulation pulses increases the nerve impulses of the upward reticular activating system (8, 9). The neural conduction is improved, the electrical activity of the nerve cells is enhanced, and neurotransmitters are released as a result of the stimulation. This leads to an increase in blood flow and brain metabolism, promoting wakefulness. The stimulation frequency is 50–100 Hz, the pulse width is between 200–500 microseconds, the amplitude is adjusted to the threshold tolerated by each patient to optimize the therapeutic effect without causing discomfort, and the duration of the stimulation is based on the patient’s clinical response as assessed periodically (10, 11). Resting-state functional MRI (rs-fMRI) has recently become a valuable tool for studying residual brain function in patients with DoC. Since these patients often cannot participate in active tasks in a nuclear magnetic scanner, rs-fMRI is an ideal method to study their brain activity (12). Information regarding the structural and functional integrity of the patient’s brain is obtained through direct functional imaging (13, 14). The amplitude of low-frequency fluctuation (ALFF) indirectly reflects spontaneous brain activation through changes in the BOLD signal, indicating brain abnormalities in the resting state (15–18). An increase in the ALFF indicates heightened spontaneous neuronal activity in the corresponding brain area, suggesting that the activation of this functional area may play a compensatory role in the loss of certain cognitive functions. Conversely, reduced ALFF values suggest a diminished intensity of spontaneous neuronal activity in the corresponding brain regions. This implies that the decreased neural activity in these areas may be associated with a decline in related cognitive functions. Consequently, ALFF can be a valuable tool for investigating fundamental brain activity in various diseases and objectively assessing overall brain function. It helps quantify consciousness levels in patients with DoC, detect residual consciousness, and evaluate the outcome of consciousness in the early stage (19–22).

Despite these promising findings, the field is still developing, and larger, more comprehensive studies are needed to validate these preliminary results (23). There is also an urgent need for nuanced biomarkers to precisely capture the complexity of consciousness (24). Therefore, this study aims to explore the predictive value of ALFF in rs-fMRI for assessing the efficacy of SCS in patients with DoC. By examining the relationship between pretreatment ALFF values and subsequent changes in consciousness levels after SCS, this study seeks to enhance our understanding of the neurobiological substrates of consciousness and support the development of targeted therapeutic interventions (25), potentially improving recovery prospects and quality of life.

## 2 Materials and methods

**Figure 1:**
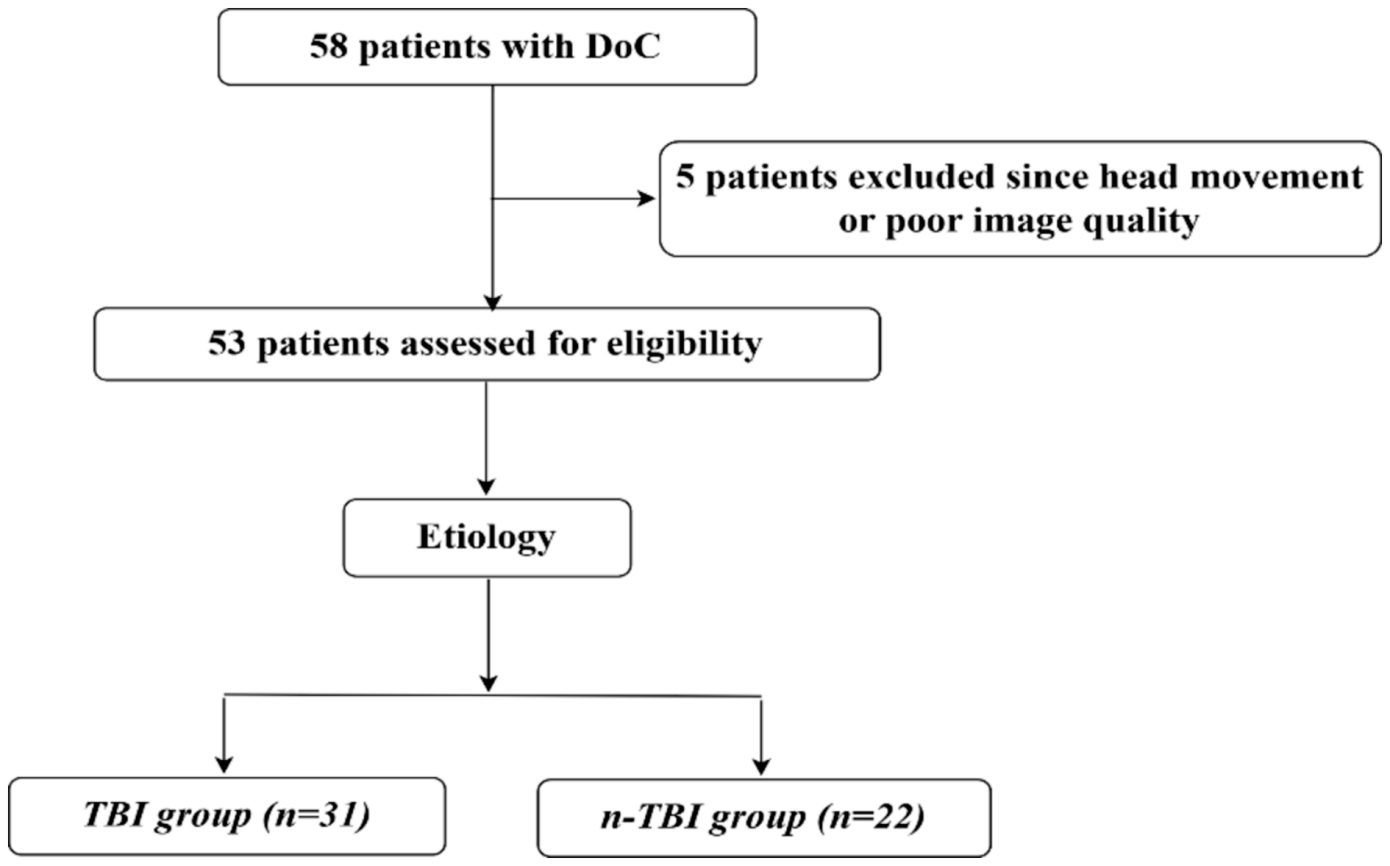
Flow chart.

Between September 2019 and November 2022, we recruited 58 patients with DoC at the Department of Neurosurgery at Peking University International Hospital. Patients’ consciousness levels were assessed preoperatively and 3 months postoperatively using the CRS-R score. Inclusion criteria were: being over 18 years old with no gender restriction; a diagnosis of DoC such as vegetative state (VS) or unresponsive wakefulness syndrome or MCS; no contraindications to MRI like metal implants or pacemakers; no sedative medication used within 24 h before the operation; and written informed consent provided by the legal guardian. Exclusion criteria included poor image quality due to excessive head movement (translation ˃3 mm or rotation ˃3°) during MRI, a small sample size, inability to complete follow-up or incomplete follow-up data, a history of psychiatric illness or severe cognitive dysfunction before the onset of the disease, and refusal of participation by legal guardians. Ultimately, 53 patients met the requirements based on the nadir criteria, and to explore whether there is a potential commonality between patients with DoC due to different aetiologies for SCS wake-up promoting treatment, they were divided into traumatic brain injury group (31 patients) and non-traumatic brain injury group (22 patients) according to the etiology. The general data for both groups are shown in Figure 1 and Table 1.

**Table 1:** General information of the two groups of patients.

Case	Diagnosis	CRS-R total score and sub-scores^a^	Etiology	Age at onset (years)	Systemic disease	Lesions (CT and/or MRI findings)	Time spent in DoC (m)	Diagnosis (3 m)	CRS-R total score and sub-scores^a^ (3 m)
1	VS	6 (1, 0, 2, 1, 0, 2)	TBI	57	None	CC, SaH, SdH	4	VS	7 (1, 1, 2, 1, 0, 2)
2	MCS^−^	10 (1, 3, 3, 1, 0, 2)	Non-TBI	69	HTN	ACA aneurysm rupture	6	MCS^−^	11 (2, 3, 3, 1, 0, 2)
3	MCS^−^	8 (1, 1, 3, 1, 0, 2)	Non-TBI	40	HTN	BGH	2	MCS^+^	13 (3, 3, 4, 1, 0, 2)
4	VS	3 (0, 0, 2, 1, 0, 0)	Non-TBI	56	CHD, MD, HTN	HI	2	VS	4 (1, 0, 2, 1, 0, 0)
5	VS	2 (0, 0, 1, 1, 0, 0)	TBI	64	HTN	CC, DAI, ImH, TL	3	VS	4 (1, 0, 2, 1, 0, 0)
6	VS	8 (1, 2, 2, 1, 0, 2)	TBI	54	None	CC, DAI, SaH, SdH	3	VS	8 (1, 2, 2, 1, 0, 2)
7	VS	6 (1, 0, 2, 1, 0, 2)	Non-TBI	47	HTN	BH	3	VS	7 (1, 1, 2, 1, 0, 2)
8	VS	7 (1, 1, 2, 1, 0, 2)	TBI	30	None	DAI, EdH, SdH, SaH	7	VS	7 (1, 1, 2, 1, 0, 2)
9	MCS^+^	10 (3, 2, 1, 2, 0, 2)	Non-TBI	18	None	AVM	4	EMCS	23 (4, 5, 6, 3, 2, 3)
10	VS	6 (1, 1, 2, 0, 0, 2)	Non-TBI	61	HTN	HI	3	MCS^+^	10 (3, 1, 3, 1, 0, 2)
11	VS	7 (1, 1, 2, 1, 0, 2)	TBI	58	DM	CC, SDH, SaH, IH	5	MCS^−^	10 (2, 2, 3, 1, 0, 2)
12	MCS^−^	10 (1, 0, 5, 2, 0, 2)	Non-TBI	48	HTN	ACA aneurysm rupture	22	MCS^+^	14 (3, 0, 5, 3, 1, 2)
13	VS	7 (1, 1, 2, 1, 0, 2)	TBI	49	None	DAI, SDH, EdH	20	VS	8 (1, 2, 2, 1, 0, 2)
14	VS	7 (1, 2, 1, 1, 0, 2)	TBI	37	None	CC, DAI, SdH	16	MCS^+^	11 (3, 3, 2, 1, 0, 2)
15	VS	6 (1, 1, 2, 1, 0, 1)	TBI	67	HTN	DAI	5	VS	5 (0, 1, 2, 1, 0, 1)
16	MCS^−^	9 (1, 3, 2, 1, 0, 2)	Non-TBI	52	HTN	BH	3	EMCS	16 (4, 5, 2, 1, 2, 2)
17	MCS^−^	8 (0, 3, 2, 1, 0, 2)	TBI	21	None	DAI, SaH	4	EMCS	20 (4, 5, 3, 3, 2, 3)
18	MCS^−^	8 (0, 3, 2, 1, 0, 2)	Non-TBI	49	DM, CHD	BH	9	MCS^−^	8 (0, 3, 2, 1, 0, 2)
19	MCS^−^	8 (0, 3, 2, 1, 0, 2)	TBI	21	None	CC, DAI, SaH, EdH	2	EMCS	18 (4, 5, 3, 1, 2, 3)
20	VS	7 (1, 1, 2, 1, 0, 2)	TBI	23	None	DAI, SdH	26	VS	7 (1, 1, 2, 1, 0, 2)
21	MCS^−^	11 (2, 3, 3, 1, 0, 2)	Non-TBI	58	HTN	BGH,	6	MCS^+^	12 (3, 3, 3, 1, 0, 2)
22	MCS^−^	9 (1, 3, 2, 1, 0, 2)	TBI	72	HTN	CC, EdH	7	VS-	6 (1, 0, 2, 1, 0, 2)
23	VS	6 (1, 0, 2, 1, 0, 2)	TBI	69	HTN, DM	CC, SdH	5	MCS^−^	9 (2, 1, 3, 1, 0, 2)
24	MCS^+^	12 (3, 4, 3, 0, 0, 2)	Non-TBI	47	CHD, DM	BI	6	MCS^−^	9 (2, 3, 2, 1, 0, 2)
25	MCS^+^	14 (3, 3, 4, 1, 0, 2)	TBI	34	None	CC, SdH	4	MCS^+^	14 (3, 3, 4, 1, 0, 2)
26	VS	6 (1, 1, 1, 1, 0, 2)	TBI	46	None	CC, DAI	6	VS	9 (2, 2, 2, 1, 0, 2)
27	VS	7 (1, 1, 2, 1, 0, 2)	TBI	57	HTN	DAI	6	MCS^−^	10 (2, 2, 3, 1, 0, 2)
28	MCS^−^	11 (2, 3, 3, 1, 0, 2)	TBI	65	HTN	BH, CC	12	MCS^−^	14 (2, 4, 5, 1, 0, 2)
29	MCS^−^	7 (0, 3, 1, 1, 0, 2)	Non-TBI	65	CHD, DM, HTN	HI	7	MCS^−^	10 (2, 3, 2, 1, 0, 2)
30	VS	7 (1, 1, 2, 1, 0, 2)	TBI	56	HTN	CC, DAI, EdH	7	VS	7 (1, 1, 2, 1, 0, 2)
31	MCS^−^	8 (1, 3, 1, 1, 0, 2)	TBI	37	None	DAI, SdH	24	MCS^+^	11 (3, 3, 1, 2, 0, 2)
32	VS	7 (1, 1, 2, 1, 0, 2)	Non-TBI	36	None	AVM	24	VS	8 (1, 2, 2, 1, 0, 2)
33	VS	6 (1, 1, 1, 1, 0, 2)	TBI	46	None	CC, EdH, SaH	6	VS	9 (2, 2, 2, 1, 0, 2)
34	MCS^−^	5 (1, 0, 3, 1, 0, 0)	Non-TBI	53	HTM	BH	3	MCS^−^	5 (1, 0, 3, 1, 0, 0)
35	VS	6 (0, 1, 2, 1, 0, 2)	Non-TBI	52	None	BGH	8	MCS^−^	7 (0, 1, 3, 1, 0, 2)
36	VS	7 (1, 1, 2, 1, 0, 2)	TBI	52	None	DAI, CC, SaH	7	MCS^−^	10 (2, 2, 3, 1, 0, 2)
37	MCS^−^	11 (1, 2, 5, 1, 0, 2)	TBI	65	HTN, DM	CC, BH	10	EMCS	19 (4, 5, 6, 1, 0, 3)
38	VS	5 (1, 0, 1, 1, 0, 2)	Non-TBI	50	HTN	ACA aneurysm rupture	7	MCS^+^	9 (3, 2, 1, 1, 0, 2)
39	MCS^−^	11 (2, 3, 3, 1, 0, 2)	TBI	32	None	DAI, BH, EdH, SaH	8	MCS^+^	14 (3, 4, 3, 2, 0, 2)
40	VS	7 (1, 1, 2, 1, 0, 2)	TBI	61	HTN	CC, EdH	5	MCS^+^	17 (4, 5, 3, 1, 1, 2)
41	VS	7 (1, 1, 2, 1, 0, 2)	TBI	36	None	CC, SaH	3	VS	8 (1, 1, 2, 1, 1, 2)
42	VS	4 (1, 0, 0, 1, 0, 2)	Non-TBI	64	HTN, DM	AVM	5	VS	4 (1, 0, 0, 1, 0, 2)
43	VS	7 (1, 2, 2, 0, 0, 2)	Non-TBI	20	None	AVM	12	VS	8 (1, 2, 2, 1, 0, 2)
44	VS	6 (1, 1, 1, 1, 0, 2)	TBI	62	HTN	CC, DAI, SaH	6	VS	6 (1, 1, 1, 1, 0, 2)
45	VS	6 (1, 0, 2, 1, 0, 2)	Non-TBI	53	HTN, DM	BH	17	MCS^+^	13 (3, 3, 3, 2, 0, 2)
46	VS	7 (1, 1, 2, 1, 0, 2)	TBI	32	None	BH, DAI	6	MCS^+^	11 (3, 3, 2, 1, 0, 2)
47	MCS^−^	8 (0, 0, 5, 1, 0, 2)	Non-TBI	30	None	BGH	11	MCS^+^	18 (3, 4, 5, 2, 1, 3)
48	VS	6 (1, 0, 2, 1, 0, 2)	Non-TBI	56	CHD, MD	BI	3	MCS^−^	10 (2, 2, 3, 1, 0, 2)
49	MCS^−^	8 (1, 2, 3, 1, 0, 1)	TBI	20	None	CC, SDH, SaH, IH	2	MCS^−^	8 (1, 3, 2, 1, 0, 2)
50	VS	4 (1, 0, 1, 1, 0, 1)	TBI	53	None	CC, DAI, SaH, EdH	1	MCS^−^	8 (3, 1, 1, 1, 0, 2)
51	VS	6 (1, 0, 2, 1, 0, 2)	TBI	64	HTN, CHD	BH, DAI, SaH	5	VS	6 (1, 0, 2, 1, 0, 2)
52	VS	3 (0, 0, 2, 1, 0, 0)	TBI	18	None	CC, DAI, SdH	6	VS	3 (0, 0, 2, 1, 0, 0)
53	MCS^−^	9 (1, 3, 2, 1, 0, 2)	Non-TBI	52	HTN	BGH	15	EMCS	16 (4, 5, 2, 1, 2, 2)

ACA, anterior communicating artery; AVM, arterial-venous malformation; BGH, basal ganglia hemorrhage; BH, brainstem hemorrhage; BI, brainstem ischemia; CC, cerebral contusions; DAI, diffuse axonal injury; EdH, epidural hematoma; HI, hemispheric ischemia; SaH, subarachnoid hemorrhage; SdH, subdural hematoma; non-TBI, non-traumatic brain injury; TBI, traumatic brain injury; MCS, minimally conscious state; VS, vegetative state; DM, diabetes mellitus; HTN, hypertension; CHD, coronary heart disease; m, month.

a CRS-R sub-scores are, in the following order: auditory, visual, motor, verbal, communication, and arousal.

After SCS, all patients underwent resuscitation and rehabilitation at our hospital and were followed up at 3 months. Follow-up was conducted via telephone for those discharged or transferred to other facilities. All patients were assessed using the CRS-R by a senior neurosurgeon specifically trained and qualified in its administration. (Items of CRS-R scale: patients in VS state show very low neurobehavioral responses on the CRS-R assessment, with scores below thresholds on all subscales: auditory ≤2, visual ≤1, motor ≤2, oral motor/verbal ≤2, and communication 0, reflecting a lack of awareness and responsiveness to the environment). Patients in the MCS state, show much higher neurobehavioral responses, with scores above the thresholds for VS: auditory 3–4, visual 2–5, motor 3–6, oro-motor/verbal 3, and communication 1–3, indicating some ability to perceive and respond to the environment, as well as more complex motor and communication behaviors. (Chart of CRS-R in the Supplementary Table S1 shows white for VS, light blue for MCS, and dark blue for EMCS).

### 2.1 fMRI data acquisition

Anatomical and functional images were collected using a Siemens 3.0 T scanner (Siemens Verio Dot 3.0 T, Germany) with an 8-channel phase-sensitivity encoding head coil (IMRIS). High-resolution T1-weighted anatomical images were acquired for each participant (TR/TE/TI = 2,300/3.25/900 ms, FA = 90°, FOV = 250 × 250 mm, image matrix: 256 × 256, 192 slices with 1-mm thickness, gap = 0 mm). Functional images were acquired using whole-brain gradient echo-planar images (TR/TE = 220/30 ms, FA = 90°, FOV = 192 × 192 mm, image matrix: 64 × 64). The scan time for wakefulness and general anesthesia was 540 s.

### 2.2 Data preprocessing

Preprocessing was performed using fMRIPrep version 21.0.2, which was built using Nipype version 1.6.1. The anatomical T1-weighted (T1w) MRI image was corrected for intensity non-uniformity and subsequent skull stripping using advanced normalization tools version 2.3.3. Brain tissue segmentation was performed to delineate the cerebrospinal fluid (CSF), white matter (WM), and gray matter (GM). The brain surfaces were reconstructed using FreeSurfer version 6.0.1. Volume-based spatial normalization was performed via nonlinear registration in a standardized space (MNI152MLin2009cAsym). The preprocessing of BOLD signals was conducted as follows. Initially, head-motion parameters, including transformation matrices and six corresponding rotation and translation parameters, were estimated relative to the BOLD reference. Subsequently, the BOLD runs underwent slice-time correction and were resampled into the original space to account for head motion, followed by co-registration of the BOLD reference with the T1w reference. Subsequently, several confounding time-series metrics were computed based on the preprocessed BOLD data, including framewise displacement (FD), DVARS (representing the rate of change in the BOLD signal across the entire brain for each data frame), and three region-specific global signals (GSs) corresponding to the CSF, WM, and entire brain mask. Physiological regressors were extracted to facilitate the component-based noise correction (CompCor). Frames exceeding a threshold of 0.5 mm FD or 1.5 standardized DVARS were identified as motion outliers. Ultimately, the BOLD time series were resampled into a standard space, resulting in a preprocessed BOLD run in the MNI152NLin2009cAsym space.

After the minimal preprocessing in fMRIPrep, several procedures were implemented in Python through the Python package Nilearn 0.9.1^1^ for further preprocessing. The BOLD time series were band-pass filtered between 0.01 Hz and 0.1 Hz, and the fMRI images were spatially smoothed using a Gaussian filter with a full width at half maximum of 6 mm. The initial five volumes of each fMRI scan were excluded from the analysis. Motion-related confounders and their derivatives (six parameters), WM, and CSF signals were regressed out. GM time series were extracted using a GM mask generated by fMRIPrep and normalized using *z*-scores to account for variance differences of non-neural origin, such as distance and head coil effects. For three patients with arterial-venous malformation (AVM)-induced DoC, the correction method entailed taking the center of the lesion area as the reference point, defining a sphere with a diameter of 3 mm, and establishing the ALFF value within the sphere as the mean value of the corresponding area within the group. This approach was adopted to minimize the impact of the AVM.

### 2.3 Temporal variability and ALFF analysis

For each participant’s state, we extracted the average time series across all voxels in the GM (i.e., the GS) and determined the with-GS SD. The same calculation was applied to the datasets after GSR, in which the SD of the GM-averaged time series was defined as without-GS SD. The voxel BOLD signals were transformed into the frequency domain using a fast Fourier transform to obtain the ALFF and the square root of the power spectrum was obtained. We calculated the SD and ALFF of each voxel in the GM and normalized the ALFF from 0 to 1. Thus, the voxel topographies of the SD and normalized ALFF were obtained. Next, we averaged the voxel-wise SD and normalized the ALFF values to obtain subject-level values. To avoid the effects of outliers, a robust regression method, random sample consensus, was applied to investigate whether GS removal altered the linear relationship between subject-level indices (with or without GS SD) and mean voxel-level indices (normalized ALFF and voxel-wise SD).

### 2.4 Evaluation criteria for consciousness improvement

The prognosis of consciousness for each patient with DoC was assessed using the CRS-R. Patients were considered to have shown improvement in consciousness if they met the following criteria: those initially classified as being in VS progressed to an MCS minus (MCS^−^), MCS plus (MCS^+^), or emerged from MCS (EMCS), or those initially classified as MCS progressed to MCS^+^ or EMCS. If the clinical diagnosis after treatment did not show improvement compared to the initial diagnosis, the clinical outcome was classified as invalid, as detailed in the Supplementary Table S1.

### 2.5 Ethics approval and consent

This study adhered to the principles of the Declaration of Helsinki and was approved by the Peking University International Hospital Review Committee (2023-KY-0023-01). Written informed consent was obtained from all patients’ legal guardians for the use of clinical data for research purposes.

### 2.6 Statistical methods

Data were analyzed using IBM SPSS Statistics software version 22.0 (IBM Corp., Armonk, NY, United States). The normality of the data was first tested using the Shapiro–Wilk test. Data that did not conform to a normal distribution were analyzed using non-parametric statistics. Comparisons between groups were made using the independent samples *t*-test or Mann–Whitney *U*-test for continuous variables and the chi-square test or Fisher’s exact test for categorical variables. Correlation analyses were performed by calculating Spearman correlation coefficients, whereas multivariate analyses employed multiple linear or logistic regression models. Statistical significance was set at *p* < 0.05.

## 3 Results

No statistically significant difference was observed in the general demographic characteristics between the two groups of patients, as shown in Tables 1, 2.

**Table 2:** Comparison of consciousness outcomes of patients with DoC in the two groups at 3 months after awakening therapy.

Parameters		TBI group (*n* = 31)		*p*	Non-TBI group (*n* = 22)		*p*
		Consciousness improved (*n* = 13)	Consciousness unimproved (*n* = 18)		Consciousness improved (*n* = 11)	Consciousness unimproved (*n* = 11)	
Preoperative diagnosis				0.197			1.00
	VS	7 (53.85%)	13 (72.22%)		5 (45.45%)	6 (54.55%)	
	MCS^−^	6 (46.15%)	3 (16.67%)		5 (45.45%)	4 (36.36%)	
	MCS^+^	0 (0.00%)	2 (11.11%)		1 (9.10%)	1 (9.09%)	
Postoperative diagnosis (3 m)				0.001			0.001
	VS	0 (0.00%)	14 (77.78%)		0 (0.00%)	6 (54.55%)	
	MCS^−^	3 (23.08%)	2 (11.11%)		2 (18.18%)	5 (45.45%)	
	MCS^+^	7 (53.84%)	2 (11.11%)		7 (63.64%)	0 (0.00%)	
	EMCS	3 (23.08%)	0 (0.00%)		2 (18.18%)	0 (0.00%)	
Systemic disease							
	HTN	4 (30.77%)	7 (38.89%)	0.718	9 (81.81%)	6 (54.54%)	0.361
	DM	1 (7.69%)	1 (5.56%)	1.000	1 (9.09%)	7 (63.64%)	0.024
	CHD	0 (0.00%)	1 (5.56%)	1.000	0 (0.00%)	4 (36.36%)	0.09
Age (year)		46.89 ± 17.44	45.77 ± 16.50	0.858	51.00 ± 13.99	47.09 ± 12.93	0.504
Sex							
	M	15 (83.33%)	12 (92.31%)	0.621	10 (90.91%)	6 (54.55%)	0.149
	F	3 (16.67%)	1 (7.69%)		1 (9.09%)	5 (45.45%)	
Time spent in DoC (m)		6 (4, 7)	5.5 (3.75, 7.25)	0.806	6 (3, 9.5)	6 (4, 10.5)	0.716

non-TBI, non-traumatic brain injury; TBI, traumatic brain injury; VS, vegetative state; MCS, minimally conscious state; EMCS, emerged from MCS; HTN, hypertension; DM, diabetes mellitus; CHD, coronary heart disease; F, female; M, male; m, month; DoC, disorders of consciousness.

### 3.1 ALFF performance in patients with improved consciousness and patients with unchanged consciousness in the TBI group

**Figure 2:**
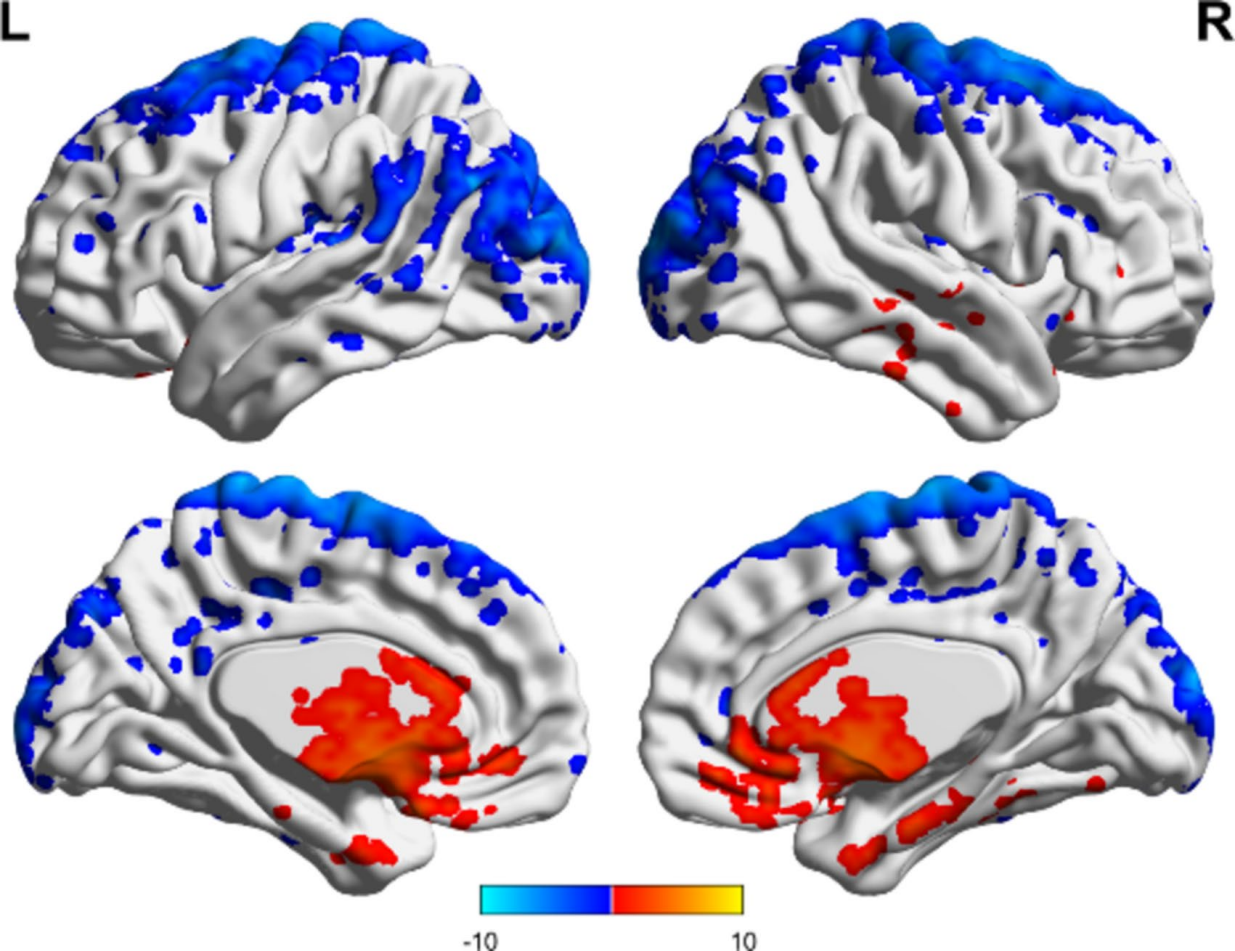
ALFF related to consciousness improvement in the TBI group. The ALFF values in the thalamus and anterior cingulate gyrus were higher than the overall average, whereas the ALFF value in the middle occipital lobe was lower than the overall average. The results are illustrated in the figure using a color-coded scale, with red indicating enhancement and blue indicating diminution.

**Figure 3:**
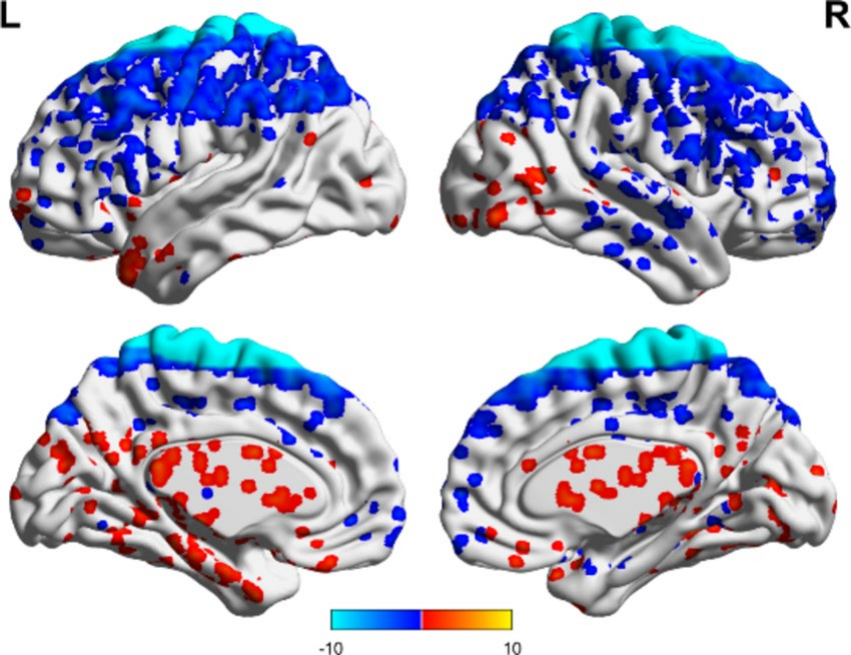
Consciousness in the TBI group did not change ALFF. No region exhibited an ALFF score that exceeded the overall average, whereas several areas demonstrated ALFF values below the overall average. These regions included the superior frontal gyrus, middle frontal gyrus, postcentral gyrus, superior posterior border gyrus, and superior lateral occipital cortex. Red indicates enhancement, while blue indicates diminution.

**Figure 4:**
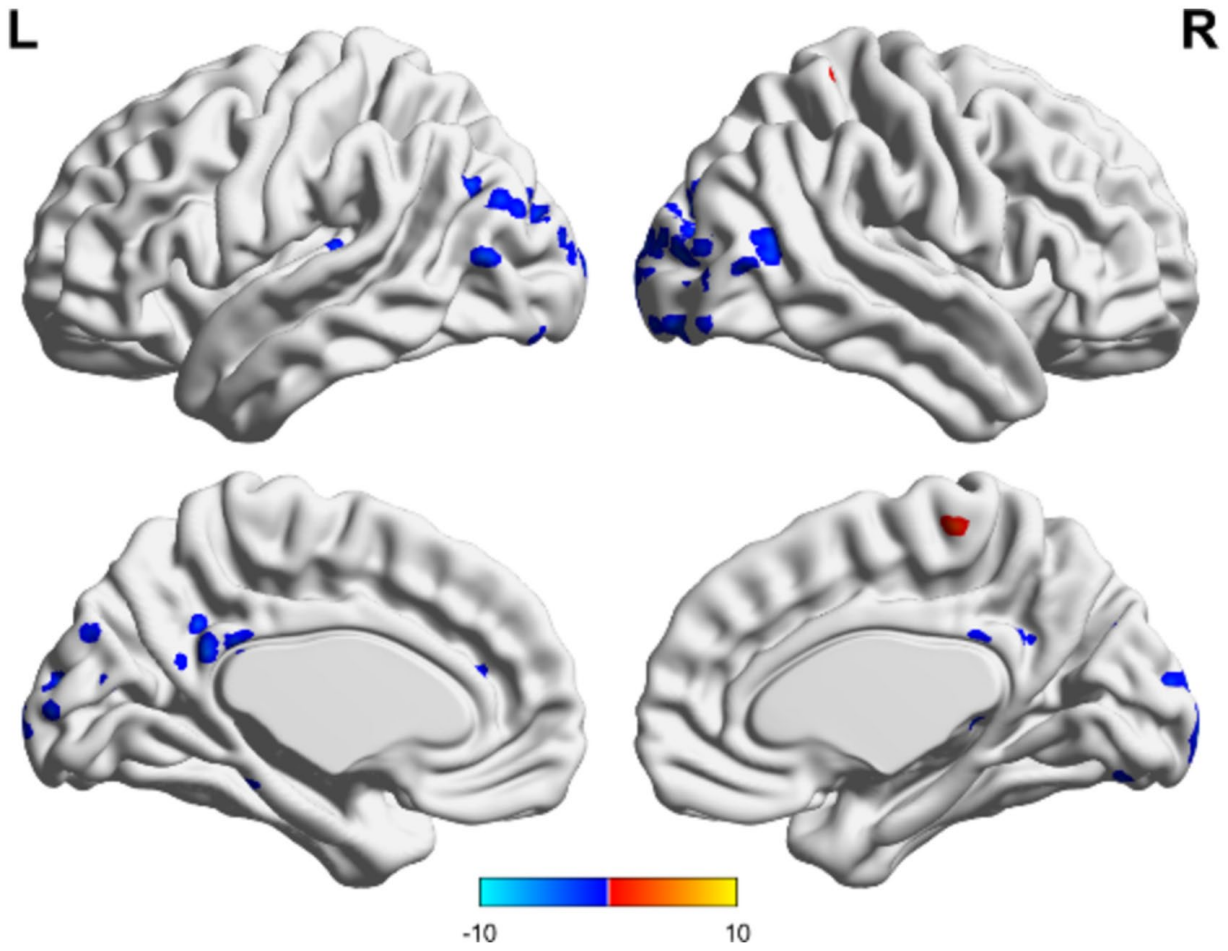
Matching comparison between unchanged consciousness and improved consciousness in the TBI group. The ALFF values were lower in the posterior cingulate gyrus and in areas that resemble the precuneus, as well as in the mid-posterior portion of the occipital lobe. These findings were observed in participants with unimproved consciousness compared to those with improved consciousness. Red indicates enhancement, while blue indicates diminution.

In the TBI group, the ALFF values in the thalamus and anterior buckle were higher than those in the entire brain, while the ALFF values in the middle occipital lobe and parietal lobes were lower than those in the entire brain. For patients with unchanged consciousness, the ALFF values were lower in the superior frontal gyrus, middle frontal gyrus, posterior central gyrus, superior posterior marginal gyrus, and superior occipital lateral cortex than in the entire brain, as shown in Figures 2, 3. Patients with improved consciousness were used as the reference for the pairing test, and it was found that the ALFF values in the posterior region close to the anterior cuneus and the middle and posterior parts of the occipital lobe in patients with unchanged consciousness were lower than those in patients with improved consciousness, as shown in Figure 4.

### 3.2 ALFF performance in patients with improved consciousness and patients with unchanged consciousness in the non-TBI group

**Figure 5:**
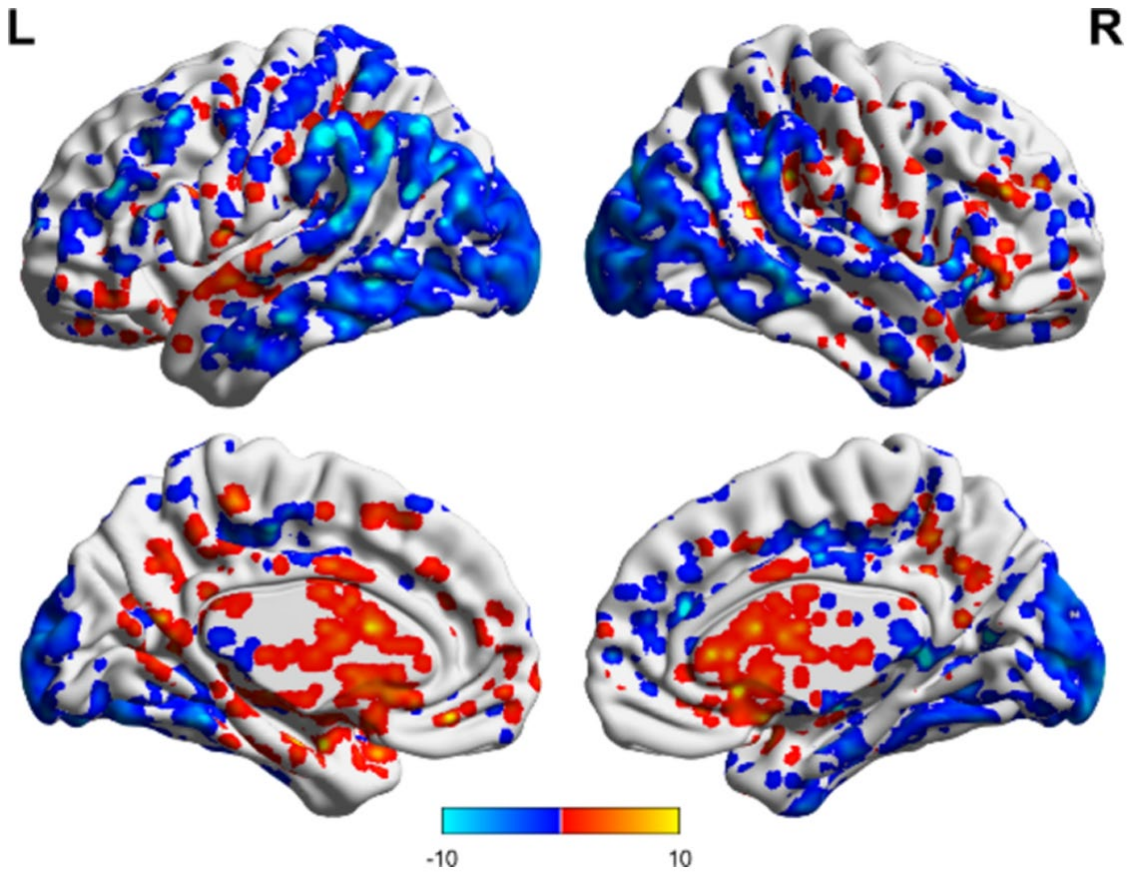
ALFF related to consciousness improvement in the non-TBI group. The ALFF values were higher in the precuneus than the overall average, whereas those in the occipital and temporal lobes were lower than the overall average. Red indicates enhancement, while blue indicates diminution.

**Figure 6:**
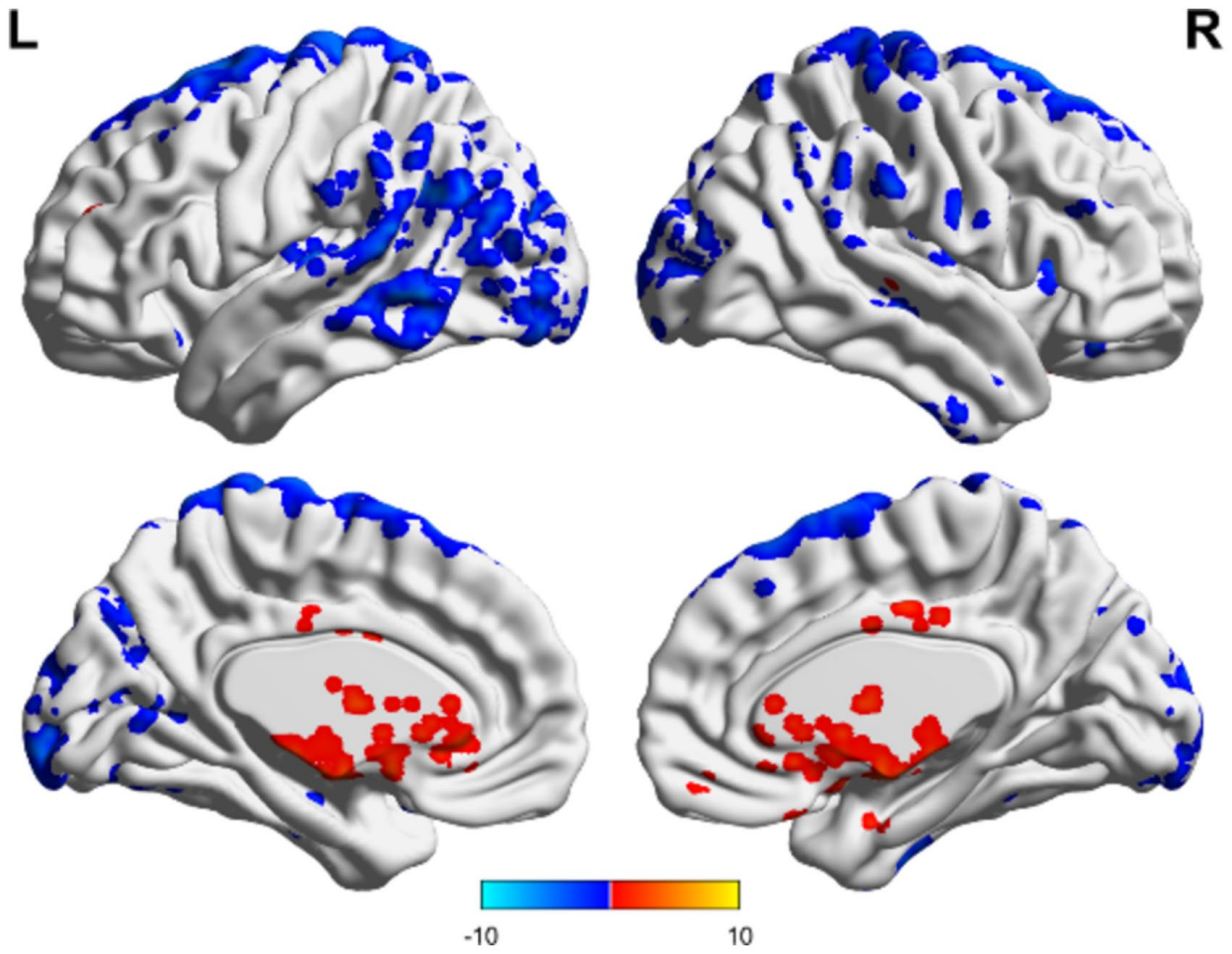
ALFF-related brain area of consciousness unchanged in the non-TBI group. No specific brain regions exhibited ALFF values exceeding the overall average, whereas motor areas of the upper frontal lobe and occipital regions demonstrated ALFF values below the overall average. Red indicates enhancement, while blue indicates diminution.

**Figure 7:**
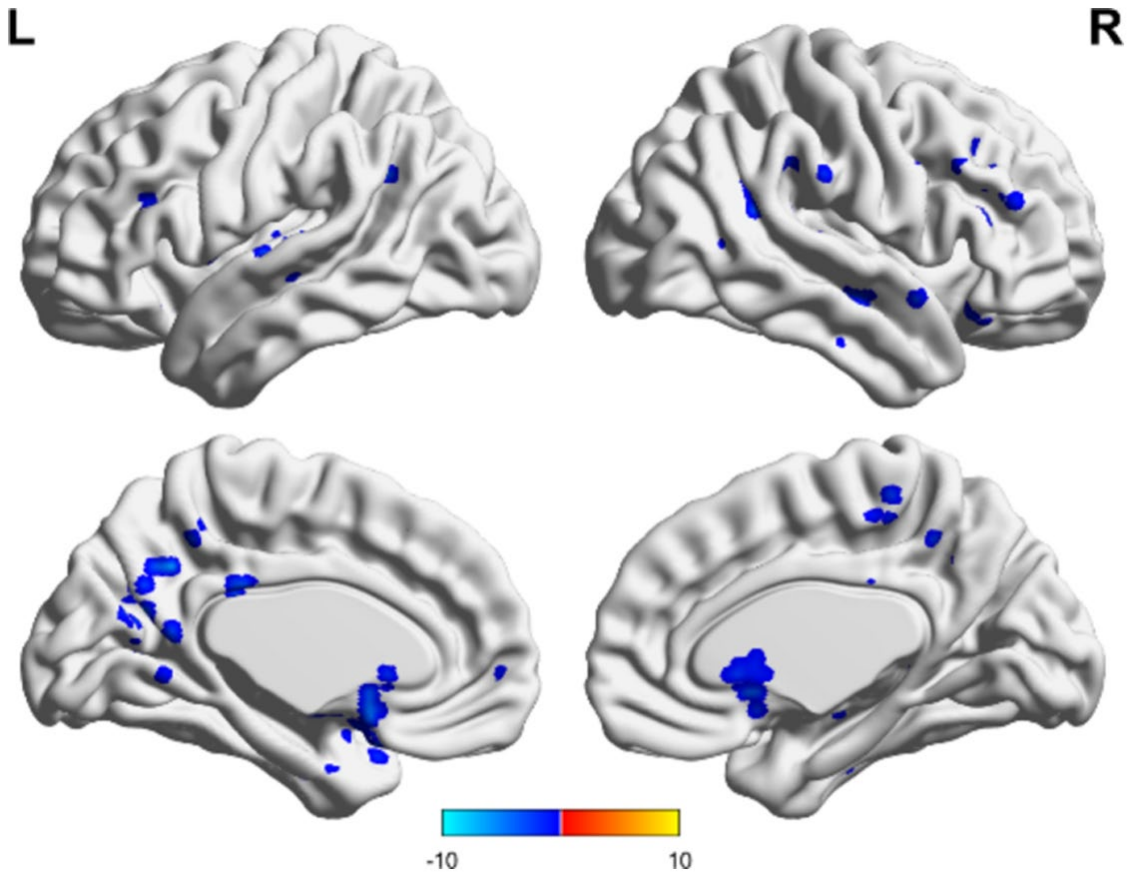
Unchanged consciousness and improved consciousness in the non-TBI group were matched. Near the posterior cingulate gyrus and precuneus, ALFF values were lower in patients with unimproved consciousness than those of patients exhibiting better levels of consciousness recovery. Red indicates enhancement, while blue indicates diminution.

In the non-TBI group, the ALFF levels in the precuneus were higher than in the entire brain, while the ALFF levels in the occipital and temporal lobes were lower than in the entire brain. For patients with unchanged consciousness, no specific brain area had ALFF values higher than the overall level, while the motor area in the upper frontal lobe had lower ALFF values compared to the entire brain, as shown in Figures 5, 6. Taking patients with improved consciousness as a reference, the ALFF value of the brain region similar to the anterior cuneus in the posterior buckle of patients with unchanged consciousness was lower than that of patients with improved consciousness, as shown in Figure 7.

### 3.3 Correlation of ALFF values in the precuneus with CRS-R scores

**Figure 8:**
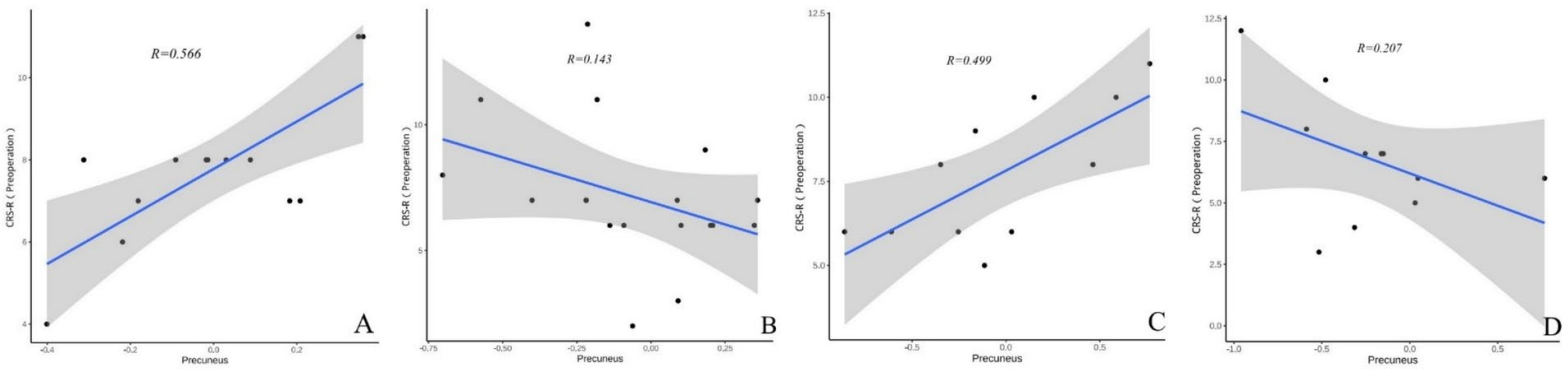
**(A–D)** Present the correlation analysis between the precuneus and the preoperative CRS-R scores of patients with different brain injuries. **(A)** Shows the correlation between the precuneus and the preoperative CRS-R scores of patients with improved consciousness in the TBI group (*r* = 0.566). **(B)** Reflects the correlation between the precuneus and the preoperative CRS-R scores of patients with unimproved consciousness in the TBI group (*r* = 0.143). **(C)** Reveals the correlation between the precuneus and the preoperative CRS-R scores of patients with improved consciousness in the non-TBI group (*r* = 0.499). **(D)** Shows the correlation between the precuneus and the preoperative CRS-R scores of patients with unimproved consciousness in the non-TBI group (*r* = 0.207).

**Figure 9:**
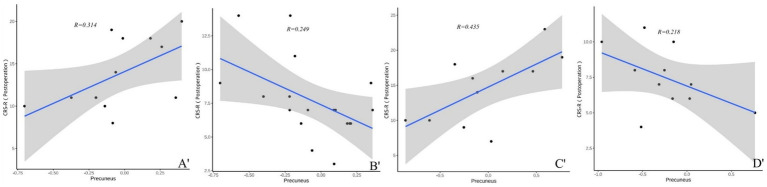
**(A′–D′)** Present the correlation analysis between the precuneus and the CRS-R scores of patients with different brain injuries 3 months after surgery. **(A′)** Shows the correlation between the precuneus and the CRS-R scores of patients with improved consciousness in the TBI group 3 months after surgery (*r* = 0.314). **(B′)** Reflects the correlation between the precuneus and the CRS-R scores of patients with unimproved consciousness in the TBI group 3 months after surgery (*r* = 0.249). **(C′)** Reveals the correlation between the precuneus and the CRS-R scores of patients with improved consciousness in the non-TBI group 3 months after surgery (*r* = 0.435). **(D′)** Shows the correlation between the precuneus and the CRS-R scores of patients with unimproved consciousness in the non-TBI group 3 months after surgery (*r* = 0.207).

In both TBI and nonon-TBI patients, the precuneus showed a strong moderate correlation with the CRS-R scores of patients with improved consciousness both preoperatively and at 3 months postoperatively (*r* = 0.566 and 0.314 VS 0.499 and 0.435) see (Figures 8, 9).

## 4 Discussion

The ALFF method in rs-fMRI was used to assess residual brain activity in patients with DoC with two etiologies and evaluate it in conjunction with the CRS-R scores and consciousness outcomes at 3 months post-SCS surgery. This approach aimed to provide insights into predicting the improvement of consciousness in patients with DoC after wakefulness-promoting treatments.

In this study, elevated ALFF values were observed in the thalamus and anterior cingulate cortex in the TBI group. The thalamus, an important brain structure linked to arousal and consciousness (26–29), is often referred to as the “on/off switch” for consciousness (30, 31). Thalamic activity levels are associated with a patient’s level of arousal (32), and decreased functional connectivity between the thalamus and cortex occurs during unconsciousness (28, 33). Damage to the thalamus and brainstem, as well as diffuse axonal injury, are the most common manifestations of DoC in autopsy and MRI studies (34–37). An increased ALFF in the thalamus may reflect the upregulation of neuronal activity in this brain region, which is a compensatory mechanism for the disruption of thalamocortical circuits (30). An increased ALFF can facilitate the reestablishment of cortical activation patterns necessary for consciousness (31), which may also be essential for the facilitation of arousal in patients with DoC. The cingulate gyrus is critical for managing cognitive and emotional functioning, overseeing conflict resolution, and processing social interactions (38–40). Increased ALFF in the cingulate gyrus may be associated with improved cognitive functioning in patients.

Interestingly, when comparing both groups of patients with DoC with those who showed improved consciousness, both groups of patients with unchanged consciousness had decreased ALFF values in the precuneus and cingulate gyrus. Conversely, both groups of patients with improved consciousness had increased ALFF in these brain regions. The precuneus, located on the medial side of the superior parietal lobule and hidden by the medial longitudinal fissure, plays a crucial role in restoring consciousness. Increased ALFF in the precuneus is associated with improved consciousness. The precuneus’s degree of centrality positively correlates with consciousness recovery in DoC patients, especially when comparing TBI and non-TBI cases, supporting this study’s findings (41). Our study aligns with existing research highlighting the precuneus’s key role in higher cognitive functions. Ionta’s et al. (42) work shows its extensive connectivity to the self-awareness network, especially between the right temporal-parietal junction and the insula, which are highly sensitive to self-positioning and first-person perspective changes. Cavanna and Trimble’s (43) anatomical and MRI studies confirmed the precuneus lobe’s activation during self-related tasks, especially in visuospatial activities and action attribution. These findings highlight the precuneus’s essential role in integrating sensory information and self-awareness, providing a crucial neurobiological basis for cognitive neuroscience and neuropsychiatric disorder treatment. The precuneus plays a central role in consciousness-related functional network aspects. It is a part of the default mode network (DMN) (41), which is associated with consciousness (43). Deficits in consciousness are closely related to the functional dissociation of the DMN, which plays an important role in the origin of consciousness (36, 44, 45). Previous studies have found that comatose patients retain spontaneously synchronized DMN activity (46, 47), suggesting that the different brain regions involved in the DMN may be correlated with the degree of improvement in consciousness. Elevated ALFF values in the precuneus may imply that DMN-connected brain regions are reactivated, which is crucial for self-awareness recovery and environmental interaction and may be a critical point for consciousness recovery (44). Notably, the precuneus is also among the earliest brain regions to show signs of recovery in patients with DoC with improved consciousness (45). A moderate positive correlation was identified between ALFF values and CRS-R scores in the precuneus. This finding suggests that the intensity of local neural activity in the precuneus may be directly correlated with the level of consciousness in patients with DoC. Increased ALFF values, which reflect the intensity of local brain activity, may indicate enhanced neural activity in this region. In the context of impaired consciousness, this increased neural activity could be associated with improvements in the patient’s level of consciousness. A significant decrease in the functional activity of the precuneus indicates an increased degree of impaired consciousness (1). This study found that patients with DoC from both etiological groups who showed no improvement in consciousness after 3 months of SCS treatment had significantly lower ALFF values in both the precuneus and cingulate gyrus. This may account for the poor improvement in consciousness observed. One possibility is that ALFF acts as a proxy for the intrinsic functional connectivity of the brain, which is often disrupted in the brain networks of patients with DoC (1). Improved functional connectivity might facilitate the reintegration of neural networks required for consciousness, as suggested by increased ALFF (48). Brain plasticity may also play an important role in this process. Neuroplasticity allows for the reorganization of brain circuits following injury and increased ALFF in key regions may indicate that these regions are undergoing adaptive changes, potentially facilitating the recovery of consciousness. The biological relevance of ALFF lies in its capacity to elucidate the intensity of spontaneous neural activity in specific brain regions. In patients with DoC, an increase in ALFF may indicate enhanced neural activity in specific brain regions, potentially representing a compensatory mechanism for loss of cognitive function. For example, in the present study, patients with DoC associated with TBI exhibited increased ALFF in the thalamus and anterior cingulate cortex, which are areas strongly associated with arousal and consciousness. An increase in ALFF may reflect enhanced neural activity in these regions, contributing to the re-establishment of cortical activation patterns, which is essential for facilitating arousal in patients. Conversely, a reduction in ALFF may indicate a decline in neural activity in the relevant brain regions, which is associated with a deterioration in cognitive function. In this study, decreases in ALFF were observed in the occipital and temporal lobes of patients with DoC. These regions are typically associated with the processing of sensory input and language functions (49, 50). The diminished activity of these regions may be associated with the sensory and cognitive impairments observed in patients. The potential of ALFF as a biomarker is based on its to predict the response to SCS therapy in patients with DoC. Notably, the present results indicate that patients whose level of consciousness improved following treatment exhibited disparate ALFF patterns in the posterior cingulate cortex and occipital lobe regions compared to those whose conditions remained unchanged. This result indicates that ALFF not only reflects the current state of neural activity but may also predict the patient’s response to treatment and the potential trajectory of consciousness recovery.

Despite these insights, our study was limited by its relatively small sample size and short follow-up period, which may affect the generalizability of our findings. Future studies with larger cohorts and long-term follow-ups are required to confirm these preliminary results. Moreover, while ALFF measures regional neural activity, it does not directly assess cognitive function or connectivity. Combining ALFF with other neuroimaging techniques, such as functional connectivity analysis or diffusion tensor imaging, may provide a more comprehensive understanding of the neural substrates of consciousness and cognition in patients with DoC. Additionally, The absence of a control group in this study limits the ability to definitively that the observed improvement in consciousness was solely attributable to SCS therapy, rather than the natural recovery of the patients or other factors. For patients with impaired consciousness who did not receive SCS therapy, obtaining clinical information, such as MRI and CRS-R scores, was challenging as they were typically managed at home. To enhance the precision of the findings, future studies should include MRI data from the same patients with impaired consciousness before and after SCS therapy (removal of SCS electrodes) to account for potential confounding factors.

In conclusion, our study suggests that ALFF measured using rs-fMRI is a promising biomarker for assessing cognitive function and levels of consciousness in patients with DoC. The observed correlation between the ALFF and consciousness highlights the potential of neuroimaging techniques in guiding therapeutic interventions and predicting outcomes for these patients. Further studies are needed to elucidate the mechanisms underlying the association between ALFF and cognitive recovery and to identify indicators of neural activity that predict the key brain regions closely associated with the recovery of consciousness in patients with DoC.

## Data Availability

The raw data supporting the conclusions of this article will be made available by the authors, without undue reservation.

## Footnotes

^1^ https://nilearn.github.io/
