# Supplementary material for: Differential brain activity in patients with disorders of consciousness: a 3-month rs-fMRI study using amplitude of low-frequency fluctuation
Source: Front Neurol. 2024 Dec 13;15:1477596. doi: 10.3389/fneur.2024.1477596 (PMC11673223; doi:10.3389/fneur.2024.1477596)
Supplement: Supplementary file 1 [file Table_1.docx]

**Supplemental material:**

1. CRS-R Scale Items

**Table 1.** CRS-R Scale Items.

| **Auditory** |  | **Visual** |  | **Motor** |  | **Promotor/verbal function** |  | **Communication** |  | **Arousal** |  |
| --- | --- | --- | --- | --- | --- | --- | --- | --- | --- | --- | --- |
| Consistent movement to command | 4 | Object recognition | 5 | Functional Object use | 6 | Intelligible verbalization | 3 | Functional: Accurate | 2 | Attention | 3 |
| Reproducible movement to command | 3 | Object localization: Reaching | 4 | Automatic motor response | 5 | Vocalization/Oral movement | 2 | Non-functional: Intentional | 1 | Eye-opening without stimulation | 2 |
| Localization to sound | 2 | Pursuit eye movements | 3 | Object manipulation | 4 | Oral reflexive movement | 1 | None | 0 | Eye-opening with stimulation | 1 |
| Auditory startle | 1 | Fixation | 2 | Localization to noxious stimulation | 3 | None | 0 |  |  | None | 0 |
| None | 0 | Visual Startle | 1 | Flexion withdrawal | 2 |  |  |  |  |  |  |
|  |  | None | 0 | Abnormal posturing | 1 |  |  |  |  |  |  |
|  |  |  |  | None | 0 |  |  |  |  |  |  |

| VS | MCS | EMCS |
| --- | --- | --- |
